# Characterization of metal oxide gas sensors via optical techniques

**DOI:** 10.1007/s00216-020-02705-6

**Published:** 2020-06-16

**Authors:** Johannes Glöckler, Carsten Jaeschke, Erhan Tütüncü, Vjekoslav Kokoric, Yusuf Kocaöz, Boris Mizaikoff

**Affiliations:** Institute of Analytical and Bioanalytical Chemistry, Ulm University, Albert-Einstein-Allee 11, 89081 Ulm, Germany

**Keywords:** Metal oxide sensor, MOX, Substrate-integrated hollow waveguide, iHWG, Fluorescence sensor, Gas sensors, Infrared sensors, Methane, Carbon dioxide, Oxygen

## Abstract

Metal oxide (MOX) sensors are increasingly gaining attention in analytical applications. Their fundamental operation principle is based on conversion reactions of selected molecular species at their semiconducting surface. However, the exact turnover of analyte gas in relation to the concentration has not been investigated in detail to date. In the present study, two optical sensing techniques—luminescence quenching for molecular oxygen and infrared spectroscopy for carbon dioxide and methane—have been coupled for characterizing the behavior of an example semiconducting MOX methane gas sensor integrated into a recently developed low-volume gas cell. Thereby, oxygen consumption during MOX operation as well as the generation of carbon dioxide from the methane conversion reaction could be quantitatively monitored. The latter was analyzed via a direct mid-infrared gas sensor system based on substrate-integrated hollow waveguide (iHWG) technology combined with a portable Fourier transform infrared spectrometer, which has been able to not only detect the amount of generated carbon dioxide but also the consumption of methane during MOX operation. Hence, a method based entirely on direct optical detection schemes was developed for characterizing the actual signal generating processes—here for the detection of methane—via MOX sensing devices via near real-time online analysis.

**Figure:**
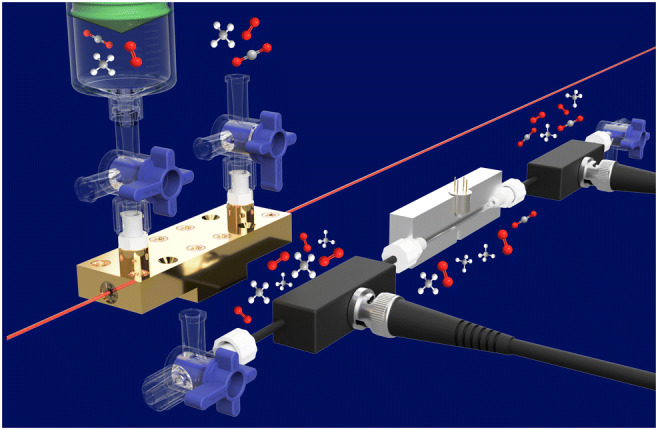
Graphical Abstract

Graphical Abstract

## Introduction

### MOX-measurement for real-time quantification of organic compounds (CH_4_)

Fast and sensitive monitoring of numerous gases at trace levels for a variety of applications has been achieved by using chemo-resistive gas sensors based on semiconducting metal oxides (MOX). Their application ranges from detecting explosive gases such as propane and toxic gases such as carbon monoxide or nitrogen dioxide [1] to targeting gas leakages at atmospheric conditions [2], and air quality sensing for volatile organic compounds (VOCs). Applications cover a wide range including agriculture [3], automotive [4], indoor air quality (IAQ) [5], and environmental gas monitoring [1]. MOX sensors have the general drawback of limited inherent selectivity [6]. The main advantages of MOX sensors systems include their small footprint, rapid response, and cost-efficiency vs. conventional analytical methods such as gas chromatography coupled to mass spectrometry (GC-MS), and Fourier transform infrared (FTIR) spectroscopy using bulky multi-pass gas cells [7, 8].

The fundamental structure of any MOX sensor comprises a substrate equipped with electrodes (e.g., ceramic Al_2_O_3_), which is coated with a sensitive layer. The electrodes enable analyzing changes in conductivity of the sensing layer. Additionally, resistance heaters are integrated, which are electrically separated by an insulating shield from the sensing later allowing for the measurement electrode to be heated up to the range of 200–400 °C [9]. Heating of the sensor layer increases the sensitivity of the MOX sensors due to the higher conductivity of the semiconductor and the faster adsorption/desorption of the target species at/from the surface. The sensor signal of MOX sensors is based on the reaction triggered by the surface of the semiconducting metal oxide and the molecular components of the sample gas. In brief, at the surface of the semiconducting metal oxides, adsorption, desorption, and interaction of gaseous components with previous adsorbed oxygen species result in a change in charge carrier concentration, which in turn affects the electrical conductivity of the material [1].

Adsorbed oxygen plays a key role in the fundamental sensing principle, as its presence enables the reduction/oxidation of target gases [10]. Although research on the mechanisms involved at the MOX surface has been going on for decades, they remain not yet fully understood and there is disagreement on the molecular details in the scientific literature [11]. One of the major problems is that many studies are carried out in synthetic environments, and therefore, the results may not be compared with realistic (i.e., real world) sensing conditions [12]. In order to overcome this limitation, studies at *in operando* conditions were performed using multi-sensor arrangements [13]. In addition to X-ray methods [14], optical methods such as UV/Vis spectroscopy [15], Raman spectroscopy [16], and diffuse reflectance infrared (DRIFT) spectroscopy [17, 18] were used to potentially detect the present surface species. Also, FTIR spectroscopy was combined with surface sensing techniques to investigate the oxidation of the analyte in the gas phase [19].

Studies on SnO_2_ surfaces have shown that the oxidation of methane takes place via several intermediate stages over acetate until complete oxidation to water and carbon dioxide. Acetaldehyde could be detected both on the surface and in the gas phase [19, 20]. However, these studies also show that these reactions do not occur in the same way on all SnO_2_ surfaces.

The surface structure and dopants [21, 22] along with the layer thickness [23] play an important role in the surface processes. Therefore, it is important to obtain detailed structure–function relationships. The temperature has probably the major influence on the type of absorption and other occurring surface reactions [24]. Studies show that already the initial adsorption of oxygen is significantly determined by the temperature of the MOX surface [25]. Also, humidity plays a significant role and has a reducing effect. For example, the reduction of SnO_2_ by humidity directly correlates to the formation of surface hydroxyl groups [26]. The target molecules also react with lattice oxygen [18], which renders it difficult to study the change in resistance as a function of oxygen concentration. In contrast to previous *in operando* studies, the present approach devises a rather simple and low-cost strategy to investigate the characteristics of commercially available or newly developed MOX sensors, rather than only MOX raw materials, i.e., substrates and powders. This design allows studying the behavior of MOX sensors while in their housings and at fully installed conditions, yet, in contrast to existing *in operando* systems without direct surface observations.

The recorded MOX sensor signal reflects a change in sensing layer resistance, which is caused by the oxidation of methane via previous adsorbed oxygen species at the surface in a rather complex mechanism ultimately forming carbon dioxide (CO_2_) and water (H_2_O). In the present study, for characterizing this behavior, we show that the resulting carbon dioxide along with the remaining methane may readily be detected using infrared spectroscopy.

### IR-measurement for real-time quantification of CH_4_ and CO_2_

In contrast to the „gold standard“ methods in gas sensing (e.g., gas chromatography coupled with mass spectrometric detection), optical techniques such as infrared spectroscopy (IR) offer a viable alternative enabling non-destructive, molecularly selective, sensitive, and close to real-time detection or monitoring of the involved molecular components [27].

With the introduction of Fourier transform infrared (FTIR) spectrometers, the measurement time of IR spectra covering the entire mid-infrared (MIR; 3–12 μm) window has been significantly reduced to few seconds, while the spectral quality has been improved to a level enabling to resolve even rotational fine structures [28]. Nowadays, IR spectroscopic techniques are a pillar of modern analytical chemistry established in a wide variety of research areas including biomedical diagnostics, industrial monitoring, and environmental analysis [29]. A main advantage of FT-MIR spectroscopy is the inherently possible identification and quantification—by using calibration methods—of almost any kind of organic and inorganic IR active molecule via their fingerprint absorptions in the MIR readily applicable to gaseous, liquid, and solid samples [18–20].

For gas-phase sensing, three main optical components are needed: a light source, a gas cell, and a detector. The most common method for FTIR is using a broad-band IR light sources with an interferometer. Common to all IR spectrometers, the excitation of specific vibrational, ro-vibrational, and rotational transitions in the MIR leads to highly molecular selective information with potential for label-free molecular diagnostics [30]. Depending on the application, a suitable detector has to be selected based on the required limit of detection (LOD). Liquid nitrogen–cooled mercury–cadmium–telluride (HgCdTe, MCT) semiconductor detectors are among the most commonly applied broad-band photodetectors offering exceptionally high LOD and a wide spectral detection range, thus frequently used in FTIR spectrometers [31]. As the gas cell, conventional vapor-phase IR technology uses multi-pass cells with extended absorption path lengths (up to several tens of meters). Such cells enable sufficient interaction between photons and molecules for maximizing the LOD; however, the probed gas volume usually exceeds few hundreds of milliliters up to several liters, which in turn yields extended sample transient time. Hence, while enabling exceptionally sensitive measurements, multi-pass gas cells are of limited utility in sensing scenario real-time analysis and thus minute probed gas volumes [32].

In order to overcome the volume limitations of multi-pass gas cells, hollow waveguides (HWG) were introduced [33]. HWGs consist of hollow silicon dioxide, sapphire, polymer, or glass tubes coated on the inside with an IR-reflective material, and serve simultaneously as an optical waveguide and as a low-volume gas cell with adequately short transient times. However, they lack in mechanical and optical robustness, flexibility, and compactness. A new generation of hollow waveguides was pioneered by the research team of Mizaikoff and collaborators, the so-called substrate-integrated hollow waveguide (iHWG). Hereby, the light-guiding channel is integrated into a solid substrate material (e.g., brass, aluminum, plastic, etc.) that simultaneously establishes the miniaturized gas cell ensuring both robustness and a small sample volume. The optical in-/outcoupling facet of the channel is sealed with IR-transparent windows (e.g., BaF_2_). Since the waveguiding channel/gas cell is integrated into a solid substrate material, the entire waveguide is mechanically extremely robust, thereby reducing optical misalignment by vibrations and eliminating mechanical stress or change in light propagation properties by bending, etc. [32]. Like conventional HWGs, iHWGs enclose a tiny gas volume (i.e., few hundreds of microliters) and provide a well-defined absorption path length. Besides, different geometries of the channel enable increasing the absorption path length while maintaining a small device footprint, thus maintaining short detection times and high temporal resolution [34]. iHWGs are compatible with a wide variety of light sources including not only coupling to entire broad-band infrared spectrometers but also to latest IR light source technologies including interband cascade lasers (ICL) and quantum cascade lasers (QCL). Since their introduction in 2013, their unmatched versatility has rendered iHWGs a key component in a wide variety of gas sensing scenarios probing species including to date ^12^CO_2_, ^13^CO_2_, CH_4_, C_4_H_1_0, H_2_S, SO_2_ NO, N_2_O, NO_2_, and ozone—individually and in mixtures—as shown by the team of Mizaikoff in combination with different light sources, detectors, and channel designs [32].

### O_2_-measurement for real-time quantification of analytes

Similar to volatile organics, the detection of oxygen concentrations plays an equally important role in a variety of application scenarios including environmental monitoring, chemical or medical analysis (e.g., oxygen concentration in blood or exhaled breath), and biotechnology [35]. Molecular oxygen is optically detected via dynamic quenching effects exerted by collision with and energy transfer from excited fluorophores, thereby enabling measurements of the accordingly reduced luminescence intensity, lifetime or shift in phase angle with increasing oxygen concentration [26, 27]. For sensing applications, luminescence intensity measurements are limited in accuracy by fluctuations of the light source, and potential degradation or leaching of the dye [36]. These effects are minimized when operating such sensors in the time domain, as luminescence lifetime is an intrinsic property of the fluorophore and independent of intensity fluctuations or detector sensitivity [37]. Even more advantageous are phase modulation techniques, whereby after excitation with sinusoidal modulated light, the luminescence lifetime may be directly correlated with a phase shift between excitation and luminescent light. Using sinusoidal modulation, fluorescence light emitted by the dye is equally modulated, and the oxygen-sensitive phase shift may be related to the lifetime and oxygen concentration [36, 38]. Packaging such sensors via fiberoptics enables remote sensing in hard-to-reach locations, over long distances, and in harsh environments [39].

The present study takes advantage of a combination of optical detection techniques—IR and luminescence sensing—for characterizing the behavior of methane-specific MOX sensors via real-time quantification of O_2_, CH_4_, and CO_2_.

## Materials and methods

### Experimental setup

The selected TGS2611-C00 MOX sensor is a semiconducting thick-film SnO_2_ metal oxide sensor designed to detect flammable gases in air. According to the data sheet of the manufacturer, the sensor TGS2611 has high sensitivity to methane, propane, and butane with similar sensitivity. These properties are determined by the characteristics of the SnO_2_ surface and the presence of dopants working as catalysts. As the precise structure of the TGS2611 sensing surface is not known, it is not possible to give precise information on the surface reactions taking place, which are essentially determined by the surface composition and structure.

The heating material on the back is RuO_2_, the lead wires are Pt–W alloy, and the connections to the sensor substrate are Ni–Fe (50%) pins. SnO_2_ is a wide bandgap n-type semiconductor, its conduction type being related to the intrinsic oxygen vacancies. SnO_2_ is used as sensing material due to its high sensitivity and stability to reducing atmospheres, while its disadvantages are low selectivity and moisture dependency [40].

**Fig. 1:**
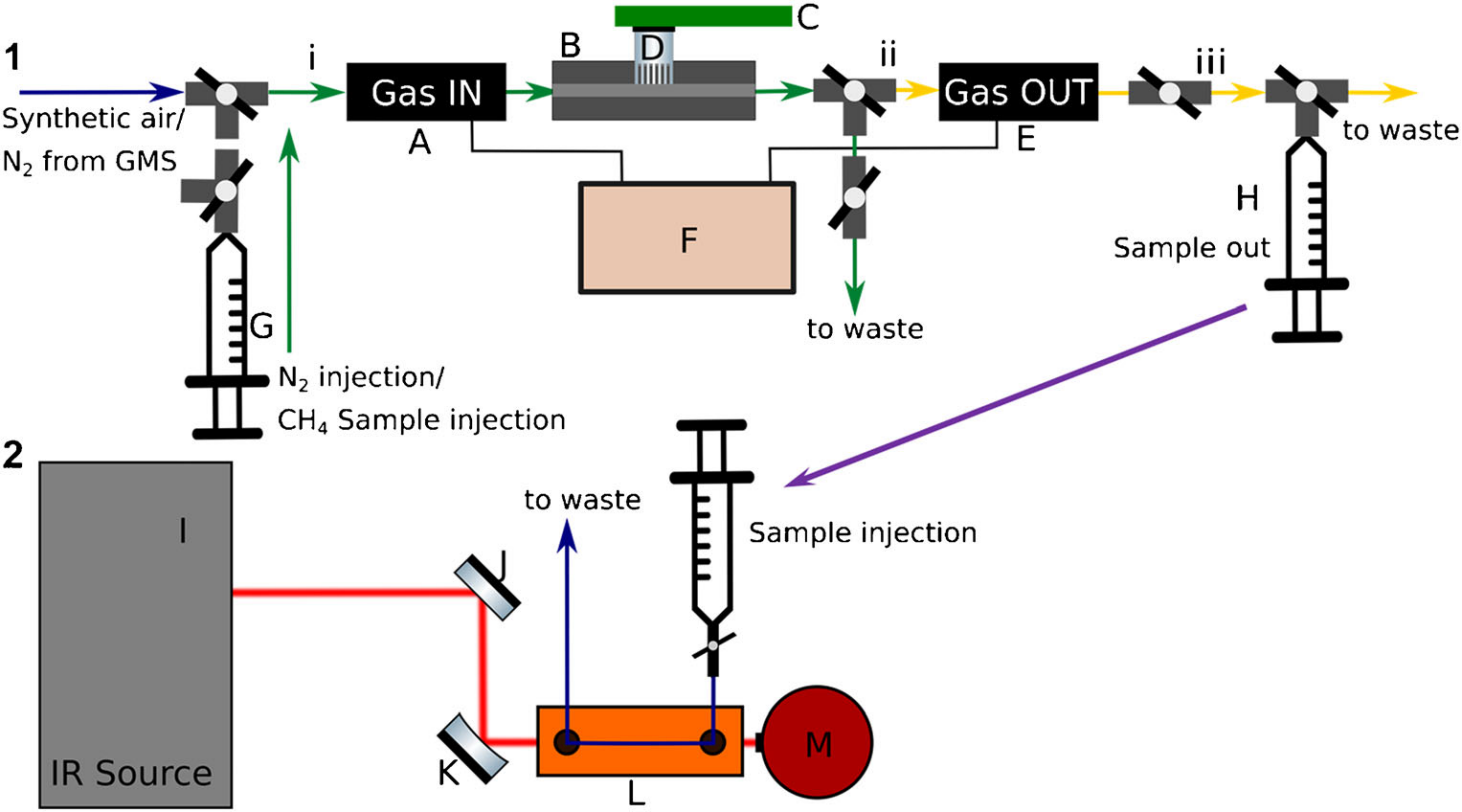
Schematic illustration of the developed 2-step approach for analyzing gaseous species during MOX operation based on direct optical detection via iHWG-coupled IR spectroscopy and luminescence-based sensors

The layout for the stopped-flow measurements is shown schematically in Fig. 1. It consists of a MOX gas sensor TGS2611-C00 (D) (Figaro Engineering Inc., Mino, Osaka, Japan) [40] which was pinned onto the USB interface MOXstick (C) (JLM Innovation GmbH, Tübingen, Germany), inserted gas-tight into the MOX gas flow-through cell (B) and sealed with one layer of Teflon tape. The sensor was controlled by JLMlogSP software (Version 2.5, JLM Innovation GmbH, Tübingen, Germany). Two fiber-optic gas flow-through fluorescence oxygen sensors OXFTC2 (Pyro Science GmbH, Aachen, Germany) were directly connected prior (A) (“Gas IN”) and after (E) (“Gas OUT”) the MOX flow-through cell (B). The polished end of the bare optical fiber (SPFIB-Bare, 1 m, Pyro Science GmbH, Aachen, Germany) was inserted into the sensor and connected by its ST-plug to the fiber-optic oxygen meter Firesting-O2 (F) (Pyro Science GmbH, Aachen, Germany). The control of oxygen sensors was enabled by Firesting Logger V2 software (Pyro Science GmbH, Aachen, Germany).

**Fig. 2:**
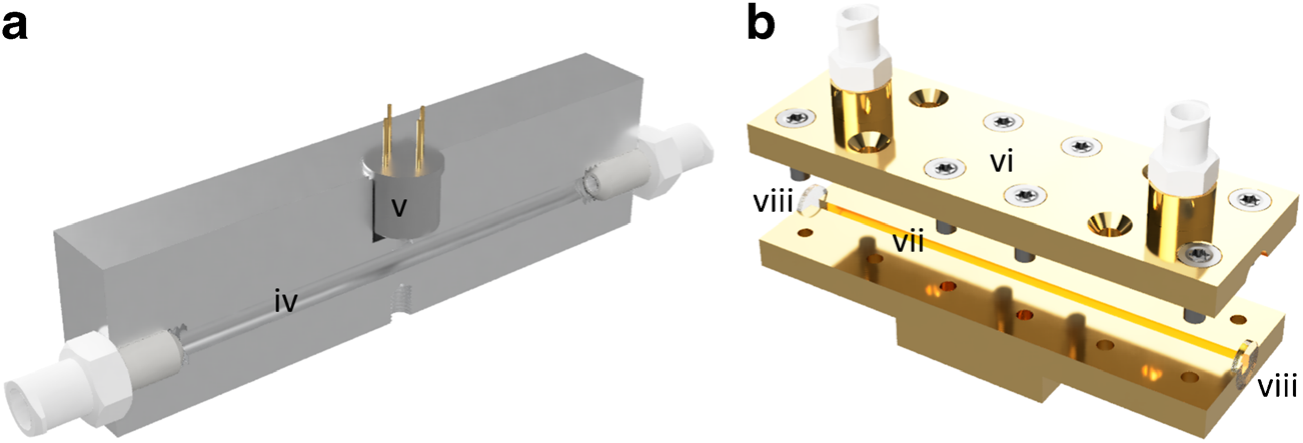
Cross-sectional view of **a** the MOX gas cell with gas channel (iv) and MOX sensor (v), and **b** an open iHWG with top substrate (vi), base substrate (vii) including the gas/light propagation channel, and BaF_2_ windows (viii)

In order to provide for a compact and robust gas cell, the entire MOX gas cell was manufactured from a 75 × 20 × 20 mm (L × W × H) aluminum block, as shown in Fig. 2a. This material offers low weight, high thermal conductivity, and robustness against oxidation. The integration of the 75-mm-long gas channel was carried out by drilling a hole through the block with a diameter of 3 mm resulting in an internal volume of 600 μL. A slot for a standardized TO-5 housing was fabricated to reach into the gas channel accommodating the MOX sensor TGS 2611-C00. Access from the MOX sensor to the gas channel is provided through a small channel with a diameter of 3 mm. This configuration of the gas path to the MOX is selected to minimize the cooling effect of the gases potentially reducing the sensitivity of the sensor during flow tests.

Precise oxygen monitoring via the optical oxygen meter was enabled by coupling an external temperature sensor for temperature compensation of the oxygen measurement. The applied oxygen sensor is based on phase modulation based on emission in the near infrared (NIR).

CO_2_ and CH_4_ were monitored using a FTIR spectrometer (I) (IRcube, Bruker Optic GmbH, Ettlingen, Germany). The emitted radiation was reflected by a planar gold mirror (J) onto an off-axis parabolic mirror (K) (both by Janos Technology Inc., Keene, NH, USA), and focused onto the incoupling facet of a 75-mm straight-channel iHWG (L) (IABC, Ulm University). After passing through the iHWG, the IR radiation was focused directly onto a liquid nitrogen-cooled mercury-cadmium telluride (MCT) detector (M) (InfraRed Associates Inc., Stuart, FL, USA) with an active detector element area of 4 mm^2^. The iHWG (Fig. 2b) used in this study was made from a brass alloy substrate. When assembled, its dimensions are 75 × 25 × 20 mm (L × W × H). The iHWG has a nominal absorption path length of 75 mm and consists of two main components. A 75-mm-long straight optical waveguide channel with a cross-section of 2.0 × 2.0 mm was milled into the substrate to form the base part. The corresponding cover substrate (top) has two threaded ports that serve as gas inlets and outlets to the iHWG channel. Both sections were polished to a mirror finish with commercially available diamond polishing suspensions to achieve a high surface reflectivity. In order to further improve the reflectivity, a gold layer was applied galvanically to the waveguide. To increase the adhesion between gold and substrate and to protect the brass from oxidation, an intermediate copper layer (copper layer thickness approx. 1 μm) had to be applied by galvanic coating. Both parts were screwed together and glued with epoxy to ensure a gas-tight connection. In order to obtain a miniaturized gas cell, both ends were sealed gas-tight with MIR-transparent BaF_2_ windows. The inner volume amounts to 300 μL.

### Measurement procedure

Prior to any experiment, the MOX sensor was preheated for at least 1 day under circuit conditions (circuit voltage *V*_C_ = 5.0 V and heating voltage *V*_H_ = 5.0 V, which leads to a sensor temperature of approx. 200 °C). The measuring frequency was set to 1 Hz. Spectral interferences of ambient air components were avoided by enclosing the entire optical setup in commercially available large low-density polyethylene (LDPE) polymer bags. To avoid any possible error due to the internal factory 2-point calibration of the oxygen sensors, both sensors were set to their default values (LED intensity 30%; amplification 400%; data smoothing 3; dphi = 50; pressure 1013 mbar; humidity 0%; temperature: external sensor) and calibrated with samples of different oxygen concentrations. The sensor recorded 1 signal per second and had a LOD of 41 ppm and a LOQ of 125 ppm (calculated according to IUPAC 3σ/10σ criteria).

To carry out the measurements, various methane gas samples were prepared in Tedlar gas sample bags (Dr. Ing. Ritter Apparatebau GmbH & Co. KG, Bochum, Germany) using a tailor-made mass flow controlled gas mixing system (GMS) developed at IABC, University of Ulm, in cooperation with the Lawrence Livermore National Laboratory (LLNL; Livermore, CA, USA).

As shown in Table 1, the measuring process started with flushing (see Fig. 1) (A)–(E) with synthetic air (20.5% O_2_, 79.5% N_2_, MTI IndustrieGase AG, Neu-Ulm, Germany) until a sensor resistance of *R*_S_ = 60 Ω was reached at the MOX sensor. This standardized starting value was selected to keep the MOX conditions consistent in each experiment and to allow a better comparison between methane samples. Immediately after reaching 60 Ω, the setup was flushed with 50 mL of nitrogen slowly injected from a plastic syringe (G) to remove oxygen from the measurement cells. Oxygen absorbed at the surface of the MOX sensor cannot be completely removed with this step. However, it remained below the LOD of the oxygen sensors, and to ensure the reproducibility of measurements, a defined volume was selected for flushing. This step is also critical for the sensor (E) Gas OUT to determine the residual oxygen concentration during the purge process from the reaction of methane/synthetic air mixtures at the MOX sensor and to calculate the total oxygen consumption of MOX between the Gas IN and Gas OUT sensors. The gas cell was closed by valves (i) and (ii) (nitrogen was trapped in the gas-out chamber) with valve (iii) and 20 mL of methane gas sample taken from the Tedlar gas bag by a plastic syringe were immediately injected into the chamber including the Gas IN (A) sensor and the MOX flow-through cell (B) (Fig. 1 green arrows) and trapped for reaction. After exactly 10 min, the path for gas from GMS (Fig. 1 horizontal arrows blue, green and yellow) was opened by switching the valves (i)–(iii) and the reacted methane sample was flushed through the Gas OUT (E) sensor into a glass syringe (H) with nitrogen from GMS at a flow rate of 2 mLn/min until a sample volume of 8 mL was reached.

**Table 1:** Measurement procedure for MOX stopped-flow experiments

	Condition	Gas path
Standardization	Syn. air 100 mLn/min	Horizontal arrows blue, green, and yellow
Flushing	N_2_ 50 mL from syringe G	Green and yellow arrows
Injection	20 mL methane gas	Green arrows, N_2_ trapped between ii and iii
Measurement MOX	10 min	Methane sample trapped between i and ii
Flushing	2 mLn/min	Horizontal arrows blue, green and yellow

Prior to IR measurements, the iHWG was flushed with nitrogen from GMS and background and blank measurements were performed. The 8 mL gas sample was then injected into the iHWG to record IR spectra of the reacted samples. The software package OPUS 6.5 (Bruker Optik GmbH, Ettlingen, Germany) was used for data acquisition and processing. Each IR spectrum is recorded in the spectral range from 4000 to 700 cm^−1^ with a resolution of 2 cm^−1^ using a Blackman–Harris 3-Term apodization function and averaged over 100 scans. This setup led to a LOD of 59 ppm and a LOQ of 179 ppm.

## Results

**Fig. 3:**
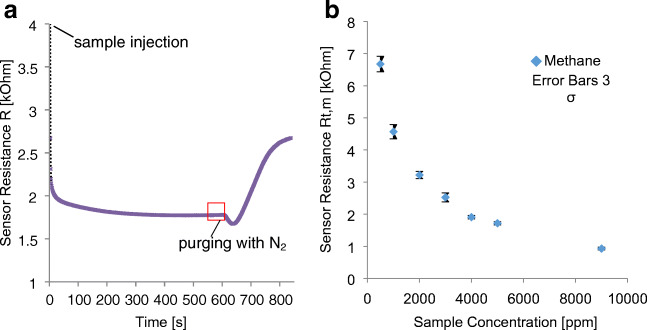
MOX sensor signal from sample injection to purging with N_2_ (**a**). Calibration function of the MOX sensor revealing the typical nonlinear behavior when plotting the mean sensor response *R*_t,m_ vs. methane concentration (**b**)

In order to study the behavior of the MOX sensor in detail, 7 different methane concentrations from 500 to 9000 ppm were analyzed for 10 min. Figure 3a shows a typical sensor signal of the MOX device. After the sample injection, the resistance drops rapidly and forms a plateau during the 10-min stopped-flow measurement phase. When purging with nitrogen, a small decrease in the sensor resistance can be observed before it rises again. For the evaluation of the sensor resistance, the last 60 s before the flushing process was used. A plot of the mean sensor resistances *R*_t,m_ versus the sample concentrations is given in Fig. 3b for obtaining a calibration function for MOX measurements from stopped-flow experiments revealing the typical nonlinear correlation. This can be explained by the saturation of the sensor surface. Desorption and adsorption are in an equilibrium, which means that additional molecules cannot absorb directly at the sensor surface providing a delayed contribution to resistance changes.

With an optimal detection range of 500–10,000 ppm specified by the manufacturer, the detection of the methane concentration in the samples was possible in an unproblematic way. However, Fig. 3b clearly shows an increase in the error bars at low concentrations. This indicates that the sensor no longer works so precisely at the lower end of the optimum range.

**Fig. 4:**
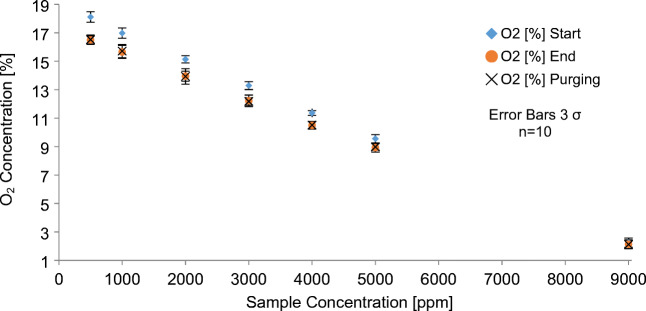
Oxygen concentrations of methane samples (i.e., sample concentration) during the reaction at the MOX sensor surface are illustrated. Values of sensor Gas IN: “Start,” “End” and Gas OUT: “Purging”

In order to quantify the consumption of oxygen and methane during these measurements, the analyzed gas was flushed out of the sampling gas cell by a flow of 2 mLn/min of nitrogen gas after the 10-min reaction time. This results in a dilution factor, which must be taken into account during subsequent measurements. This was achieved by analyzing synthetic air (20.5% O_2_, 0 ppm CH_4_) in the setup with the MOX sensor deactivated. From the O_2_ value of the Gas IN (A Fig. 1) sensor (“Start”) to the oxygen concentration registered by the Gas OUT (E Fig. 1) sensor during the purge, the dilution of the unreacted synthetic air sample was determined to 3.1%. For all methane sample measurements, this determined deviation was then added to the oxygen values of the second sensor during “purging.” This step has a synergistic effect on the analysis, as it allows the data evaluation of two and not only one sensor. The mean values of the analyzed data from the corrected oxygen values mentioned above for the Gas OUT sensor are shown in Fig. 4. The oxygen concentration is plotted against the sample methane concentration.

The difference in oxygen concentration between “Start” and “End” for all samples indicates that the oxidation of methane is associated with oxygen consumption. In a direct comparison of both sensors, the obtained data show that the determined relative dilution and the subsequent correction of the sensor Gas OUT largely correspond to the measured O_2_ values at step “End” of the sensor Gas IN. Furthermore, this result also confirms the reproducibility of the entire analysis procedure.

When investigating the oxygen measurement values from Table 2, it is noticeable that the consumption of oxygen molecules decreases with increasing methane concentration. This can be explained by the decreasing concentration of oxygen, which was present in the methane samples. This was caused by the dilution of the methane samples with synthetic air. However, this decrease of oxygen consumption while the methane concentration increases clearly shows that the consumption of oxygen by methane oxidation is convoluted by stronger effects, if enough oxygen is available. The adsorption of oxygen on the MOX surface has to be considered. With a higher concentration of oxygen, a higher percentage of oxygen adsorbs. These oxygen molecules desorb during purging of the gas chamber with nitrogen over a longer period of time. Therefore, they are not detected in this experimental setup and cause a significant decrease in the oxygen concentration in the gas phase during the reaction time.

**Table 2:** Changes in gas concentrations during MOX measurements

Concentration [ppm]	Δ CH_4_ [ppm]	Δ CO_2_ [ppm]	Δ O_2_ [ppm]
500	123	112	8075
1000	234	147	6630
2000	463	172	5915
3000	572	197	5335
4000	784	221	4340
5000	833	244	3130
9000	978	303	570

**Fig. 5:**
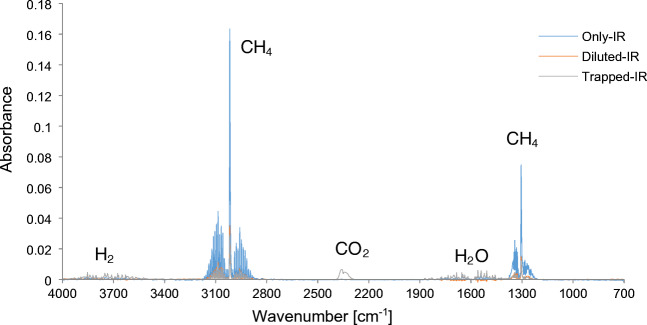
Summary of 4000 ppm CH_4_ IR measurements. “Only-IR” (blue): direct injection in iHWG. “Diluted-IR” (orange): sample in the setup with deactivated MOX, therefore only dilution and no conversion contribution. “Trapped-IR”: sample trapped for 10 min for reaction at MOX sensor surface (gray)

As already described for the oxygen measurements, the transfer of the gas sample into a glass syringe after 10 min dilutes the sample with nitrogen, thereby reducing its concentration. For further knowledge about processes that occur during the reaction, it is not sufficient to only record IR spectra of reacted samples (i.e., “Trapped-IR”), since the influences on the concentration by dilution and diffusion are unknown. Hence, likewise, methane samples in the setup while deactivating the MOX sensor were recorded leading to reference spectra of the diluted sample (i.e., “Diluted-IR”), which allows deriving the percentage change during conversion. By additional direct measurements of the specific gas samples by iHWG (i.e., “Only-IR”), the relative dilution of the samples (i.e., difference between “Only-IR” and “Diluted-IR”) and the consumption of CH_4_ ([ppm] and [%]; from “Trapped-IR” with known dilution correlation to “Only-IR”) may be calculated. An overview of the IR spectra obtained for 4000 ppm methane samples is given in Fig. 5 providing exemplary “Only-IR,” “Diluted-IR,” and “Trapped-IR” spectra. It is clearly evident that in addition to the methane signals, signals for water and CO_2_ have emerged. These signals result from the complete oxidation of the methane. However, no oxidative intermediate product can be detected. This may be due to a concentration below the LOD, as in this scenario less partially oxidized methane species are desorbed from the MOX surface. This can be influenced by the surface temperature, the surface structure, or the surface chemistry.

The integration of the CH_4_-band around 3017 cm^−1^ of all sample mixtures injected directly into the iHWG, and the samples with deactivated MOX sensor resulted in a mean value of the dilution at 79.12%. As the measurement principle of target molecules with MOX sensors is based on a reduction/oxidation process with adsorbed oxygen species at the surface, methane is essentially converted into CO_2_ and H_2_O. The drop in methane concentration can be calculated by comparing “Trapped-IR” and “Dilute-IR” spectra, as both samples are diluted during the purge process within the system. The integration of CH_4_-band for all methane sample mixtures captured for 10 min was compared with the values of “Diluted-IR.” Evidently, the oxidation of methane leads to a decrease in CH_4_ concentration, and vice versa, to an increase in CO_2_ content. Therefore, the peak area of the CO_2_ band was likewise integrated and compared for “Diluted-IR” (i.e., deactivated MOX sensor) and “Trapped-IR” (i.e., methane trapped for 10 min). Increasing CO_2_ values reveal a linear behavior (regression with *R*^2^ = 0.97) with increasing CH_4_ sample concentration.

As previously discussed, the dilution of the samples for iHWG measurements and the relative values of methane decrease/carbon dioxide increase were determined. To obtain absolute data on the methane concentration changes, the peak area of the enclosed sample was related to the concentration of the individual IR samples (i.e., undiluted and undiluted) by cross-multiplication, and subtracted from the diluted IR concentration. The following Eq. (1) correlates the peak area of the sample with the initial concentration (e.g., 1000 ppm CH_4_; Abs to absolut and IPA to integrated peak area).

1 $$ \mathrm{C}{\mathrm{H}}_4\ \mathrm{decrease}\ \left[\mathrm{ppm}\right]=\frac{1000\ \mathrm{ppm}}{\mathrm{IP}{\mathrm{A}}_{\mathrm{Only}-\mathrm{IR}}}\cdotp \left(\mathrm{IP}{\mathrm{A}}_{\mathrm{Diluted}-\mathrm{IR}}-\mathrm{IP}{\mathrm{A}}_{\mathrm{Trapped}}\right) $$

Since the mean value of the sample dilution was determined at 79.12%, the calculated values for the CH_4_ reduction at the MOX sensor represent only 20.88%. Therefore, all values were subsequently corrected to 100%. To then determine the absolute increase in CO_2_ content, a mixture of 500 ppm CH_4_ and 500 ppm CO_2_ in synthetic air was injected directly into the iHWG and analyzed. As with methane, the determined CO_2_ increase is diluted, and thus, must be corrected by cross-multiplication (i.e., from 20.88 to 100%).

Table 2 shows the changes of the measured gases during the MOX experiment. It is evident that not all of the CH_4_ consumed has been completely oxidized to CO_2_, as it is known that not always complete oxidation occurs [19]. However, the presumably formed species could not be detected in the IR spectrum, as the concentration was too low. It is also possible that CH_4_ or partially oxidized intermediate stages molecules may remain at the MOX surface, and desorb only later. In fact, the same is true for oxygen. Last but not least, it is not known how much oxygen was initially adsorbed at the MOX surface prior to starting the experiment.

**Fig. 6.**
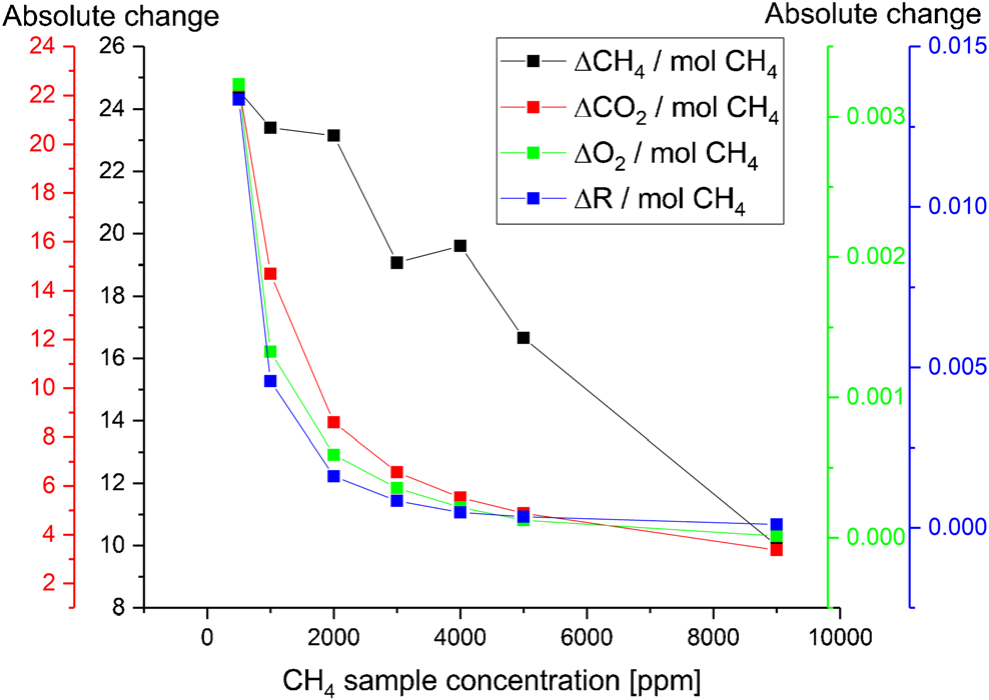
Absolute changes during a typical stopped-flow experiment

Figure 6 shows the correlation between the consumption of methane and oxygen, and the formation of CO_2_. In addition, the change in resistance during the measurements is shown. While the conversion is apparently not stoichiometric, a distinct relation is evident.

As described earlier, the oxidation of methane is occurring via a highly complex surface mechanism. While the calculated values show a proportional behavior of CH_4_ conversion into CO_2_, the methane concentration determined after the reaction still increases, as the MOX sensor has not converted all available methane.

These results confirm that the change in resistance is directly related to the adsorbed oxygen species, whereas the concentration of the species to be oxidized is not in a linear relation. This is known from the general correlation of the concentration to the resistance in Fig. 3b.

## Conclusions

In summary, it was demonstrated that simultaneous measurements in real time taking advantage of orthogonal direct optical sensing principles—infrared and luminescence—enables simultaneous methane, carbon dioxide, and oxygen sensing for characterizing the behavior of metal oxide–based semiconductor sensors during the active detection process. Taking into account dilution factors, absolute concentration values for the molecular components relevant during MOX sensor operation have been derived illustrating oxygen consumption, methane decrease, and carbon dioxide increase. The obtained data was highly reproducible in terms of sample injection, gas flow, timing, individual sensor response, and relative dilution during the purging process, which renders the developed approach suitable as a routine characterization tool for MOX sensors and electronic noses. While demonstrated for MOX sensors in the present study, of course, also other gas sensors consuming or converting species during the analytical process may be characterized.

A main shortcoming of the proposed strategy may be considered that no direct information on the processes occurring at the sensor surface is provided. Only desorbed gases are measured. Therefore, no direct statements on the actual conversion mechanisms can be made. In this measuring setup, it was not possible to detect acetaldehyde as intermediate stage from methane to fully oxidized CO_2_. Nonetheless, the observation of changes in gas composition close to real time may provide additional insight into these processes, and into the precise correlation between changes in sensor resistance and the conversion processes taking place at the MOX surface. The main advantage of the developed direct IR detection strategy is that it is a straightforward method that may not only detect gaseous species occurring during MOX operation close to real time in routine characterization, but is sufficiently robust that it may be used in mobile application scenarios enabling IR spectroscopic characterization of MOX devices at *in operando* conditions. Future experiments based on the fundamental demonstration of the feasibility of this approach will focus on humidity, which is another major parameter affecting MOX signals.

## References

[1] Fine GF, Cavanagh LM, Afonja A, Binions R (2010). Metal oxide semi-conductor gas sensors in environmental monitoring. Sensors.

[2] Wang C, Yin L, Zhang L, Xiang D, Gao R (2010). Metal oxide gas sensors: sensitivity and influencing factors. Sensors.

[3] Mitzner KD, Sternhagen J, Galipeau DW (2003). Development of a micromachined hazardous gas sensor array. Sensors Actuators B Chem.

[4] Yamazoe N (2005). Toward innovations of gas sensor technology. Sensors Actuators B Chem.

[5] Zampolli S, Elmi I, Ahmed F, Passini M, Cardinali GC, Nicoletti S, Dori L (2004). An electronic nose based on solid state sensor arrays for low-cost indoor air quality monitoring applications. Sensors Actuators B Chem.

[6] Arshak K, Moore E, Lyons GM, Harris J, Clifford S (2004). A review of gas sensors employed in electronic nose applications. Sens Rev.

[7] Nagle HT, Gutierrez-Osuna R, Schiffman SS (1998). The how and why of electronic noses. IEEE Spectr.

[8] Dey A (2018). Semiconductor metal oxide gas sensors: a review. Mater Sci Eng B Solid-State Mater Adv Technol.

[9] Barsan N, Koziej D, Weimar U (2007). Metal oxide-based gas sensor research: how to?. Sensors Actuators B Chem.

[10] Yamazoe N, Shimanoe K (2008). Theory of power laws for semiconductor gas sensors. Sensors Actuators B Chem.

[11] Gurlo A (2006). Interplay between O2 and SnO2: oxygen ionosorption and spectroscopic evidence for adsorbed oxygen. ChemPhysChem.

[12] Staerz A, Suzuki T, Weimar U, Barsan N. SnO2: the most important base material for semiconducting metal oxide-based materials: Elsevier Inc.; 2020.

[13] Gurlo A, Riedel R (2007). In-situ- und Operando-Spektroskopie zur Untersuchung von Mechanismen der Gaserkennung. Angew Chem.

[14] Koziej D, Hübner M, Barsan N, Weimar U, Sikora M, Grunwaldt JD (2009). Operando X-ray absorption spectroscopy studies on Pd-SnO2 based sensors. Phys Chem Chem Phys.

[15] Degler D, Barz N, Dettinger U, Peisert H, Chassé T, Weimar U, Barsan N (2016). Extending the toolbox for gas sensor research: operando UV/vis diffuse reflectance spectroscopy on SnO2-based gas sensors. Sensors Actuators B Chem.

[16] Sänze S, Gurlo A, Hess C (2013). Beobachtung von Gassensoren während des Betriebs: Operando-Raman-FTIR-Studie zur Ethanol-Detektion durch Indiumoxid. Angew Chem.

[17] Harbeck S, Szatvanyi A, Barsan N, Weimar U, Hoffmann V (2003). DRIFT studies of thick film un-doped and Pd-doped SnO2 sensors: temperature changes effect and CO detection mechanism in the presence of water vapour. Thin Solid Films.

[18] Degler D, Wicker S, Weimar U, Barsan N (2015). Identifying the active oxygen species in SnO2 based gas sensing materials: an operando IR spectrsocopy study. J Phys Chem C.

[19] Elger AK, Hess C (2019). Elucidating the mechanism of working SnO2 gas sensors using combined operando UV/Vis, Raman, and IR spectroscopy. Angew Chem Int Ed.

[20] Kohl D (1989). Surface processes in the detection of reducing gases with SnO2-based devices. Sensors Actuators.

[21] Schilling C, Ziemba M, Hess C, Ganduglia-Pirovano MV (2020). Identification of single-atom active sites in CO oxidation over oxide-supported Au catalysts. J Catal.

[22] Degler D, Weimar U, Barsan N (2019). Current understanding of the fundamental mechanisms of doped and loaded semiconducting metal-oxide-based gas sensing materials. ACS Sensors.

[23] Ziegler D, Palmero P, Tulliani J-M, Staerz A, Oprea A, Weimar U, Barsan N (2019). Investigation of the film thickness influence on the sensor response of In2O3-based sensors for O3 detection at low temperature and operando DRIFT study. Proceedings.

[24] Kato Y, Yoshikawa K, Kitora M (1997). Temperature-dependent dynamic response enables the qualification and quantification of gases by a single sensor. Sensors Actuators B Chem.

[25] Lenaerts S, Roggen J, Maes G (1995). FT-IR characterization of tin dioxide gas sensor materials under working conditions. Spectrochim Acta Part A Mol Biomol Spectrosc.

[26] Wicker S, Guiltat M, Weimar U, Hémeryck A, Barsan N (2017). Ambient humidity influence on CO detection with SnO2 gas sensing materials. A Combined DRIFTS/DFT Investigation. J Phys Chem C.

[27] Hodgkinson J, Tatam RP. Optical gas sensing: a review. Meas Sci Technol. 2013;24. 10.1088/0957-0233/24/1/012004.

[28] Sieger M, Haas J, Jetter M, Michler P, Godejohann M, Mizaikoff B. Mid-infrared spectroscopy platform based on GaAs/AlGaAs thin-film waveguides and quantum Cascade lasers. Anal Chem. 2016;88. 10.1021/acs.analchem.5b04144 26845392

[29] Schrader B, Bougeard D. Infrared and Raman spectroscopy : methods and applications: VCH; 1995.

[30] Kim SS, Young C, Vidakovic B, Gabram-Mendola SGA, Bayer CW, Mizaikoff B (2010). Potential and challenges for mid-infrared sensors in breath diagnostics. IEEE Sensors J.

[31] Stuart BH (2005) Infrared spectroscopy: fundamentals and applications.

[32] Mizaikoff B (2013). Waveguide-enhanced mid-infrared chem/bio sensors. Chem Soc Rev.

[33] Wilk A, Seichter F, Kim SS, Tütüncü E, Mizaikoff B, Vogt JA, Wachter U, Radermacher P (2012). Toward the quantification of the 13CO 2/ 12CO 2 ratio in exhaled mouse breath with mid-infrared hollow waveguide gas sensors. Anal Bioanal Chem.

[34] Fortes PR, da Silveira Petruci JF, Wilk A, Cardoso AA, Raimundo IM, Mizaikoff B (2014). Optimized design of substrate-integrated hollow waveguides for mid-infrared gas analyzers. J Opt.

[35] MacCraith BD, McDonagh CM, O’Keeffe G, Keyes ET, Vos JG, O’Kelly B, McGilp JF (1993). Fibre optic oxygen sensor based on fluorescence quenching of evanescent-wave excited ruthenium complexes in sol-gel derived porous coatings. Analyst.

[36] Jorge PAS, Caldas P, Rosa CC, Oliva AG, Santos JL (2004). Optical fiber probes for fluorescence based oxygen sensing. Sensors Actuators B Chem.

[37] McDonagh C, Kolle C, McEvoy AK, Dowling DL, Cafolla AA, Cullen SJ, MacCraith BD (2001). Phase fluorometric dissolved oxygen sensor. Sensors Actuators B Chem.

[38] Chodavarapu VP, Shubin DO, Bukowski RM, Titus AH, Cartwright AN, Bright FV (2007). CMOS-based phase fluorometric oxygen sensor system. IEEE Trans Circuits Syst I Regul Pap.

[39] Wang XD, Wolfbeis OS (2016). Fiber-optic chemical sensors and biosensors (2013-2015). Anal Chem.

[40] Figaro Engineering Inc. (2017) Technical Information for TGS2611 Methane Gas Sensor. 13

